# The p75 neurotrophin receptor is expressed by adult mouse dentate progenitor cells and regulates neuronal and non-neuronal cell genesis

**DOI:** 10.1186/1471-2202-11-136

**Published:** 2010-10-20

**Authors:** Ramon O Bernabeu, Frank M Longo

**Affiliations:** 1 Department of Neurology, UCSF/VAMC, San Francisco, CA, 94121, USA; 2 Institute of Cell Biology and Department of Physiology, University of Buenos Aires, Buenos Aires, Argentina; 3 Department of Neurology and Neurological Sciences, Stanford University, Palo Alto, CA, 94035, USA

## Abstract

**Background:**

The ability to regulate neurogenesis in the adult dentate gyrus will require further identification and characterization of the receptors regulating this process. *In vitro *and *in vivo *studies have demonstrated that neurotrophins and the p75 neurotrophin receptor (p75^NTR^) can promote neurogenesis; therefore we tested the hypothesis that p75^NTR ^is expressed by adult dentate gyrus progenitor cells and is required for their proliferation and differentiation.

**Results:**

In a first series of studies focusing on proliferation, mice received a single BrdU injection and were sacrificed 2, 10 and 48 hours later. Proliferating, BrdU-positive cells were found to express p75^NTR^. In a second series of studies, BrdU was administered by six daily injections and mice were sacrificed 1 day later. Dentate gyrus sections demonstrated a large proportion of BrdU/p75^NTR ^co-expressing cells expressing either the NeuN neuronal or GFAP glial marker, indicating that p75^NTR ^expression persists at least until early stages of maturation. In p75^NTR ^(-/-) mice, there was a 59% decrease in the number of BrdU-positive cells, with decreases in the number of BrdU cells co-labeled with NeuN, GFAP or neither marker of 35%, 60% and 64%, respectively.

**Conclusions:**

These findings demonstrate that p75^NTR ^is expressed by adult dentate progenitor cells and point to p75^NTR ^as an important receptor promoting the proliferation and/or early maturation of not only neural, but also glial and other cell types.

## Background

Neurons and astrocytes in the dentate gyrus of the hippocampus continue to be replaced throughout adult life in several species including humans [1-6]. Given the therapeutic implications of promoting neurogenesis in the dentate gyrus [7,8], it is becoming increasingly important to identify the mechanisms involved in the early stages of adult stem cell proliferation and differentiation. Receptors amendable to small molecule therapeutic targeting are of particular interest.

Several lines of evidence raise the possibility that neurotrophins and their receptors might be capable of regulating dentate progenitor proliferation and/or differentiation. Mature neurotrophins interact with two types of receptors: the Trk tyrosine kinase receptors (TrkA, TrkB, and TrkC) and the p75 neurotrophin receptor (p75^NTR^) [9]. p75^NTR ^receptors promote neuronal death or survival depending on the cellular context and the actions of a complex array of intracellular adaptors [10-12]. In *in vitro *studies, p75^NTR^-linked signaling has also been found to regulate cell cycle progression and/or cellular maturation of the following cell types: human oral keratinocyte stem/progenitor cells [13]; myoblasts [14], PC12 cells [15]; neuroblasts [16], embryonic striatal progenitors [17]; embryonic forebrain neurospheres [18]; and subventricular zone cells [19]. Mouse embryonic stem cells have been shown to express p75^NTR ^and NGF-induced proliferation of these cells can be inhibited by a p75^NTR ^blocking antibody [20].

The question of whether p75^NTR ^regulates proliferation or maturation of neural progenitors *in vivo *has been examined in the context of the subventricular zone (SVZ), olfactory bulb and subgranular zone (SGZ) of the dentate gyrus. Giuliani et al [21] demonstrated that p75^NTR ^is expressed by a large population of dividing cells in the adult SVZ while TrkA and TrkB expression was not detected. In NCAM-/- mice, Gascon et al. [22] found increased p75^NTR ^expression in the rostral migratory stream-olfactory bulb that was associated with early maturation and increased levels of death in the progenitor population. Young et al. [23] also found that p75^NTR ^is expressed by SVZ cells in adult mice. In p75^NTR-/- ^mice, they observed a 25-45% reduction in the number of SVZ PSA-NCAM-positive neuroblasts and a significant reduction in olfactory bulb weight. In neurospheres derived from these mice, in which expression of TrkA or TrkB receptors was not detected, BDNF- and NGF-induced neurogenesis was found to be mediated entirely by p75^NTR^, further pointing to a role for p75^NTR ^in regulating SVZ neurogenesis. A recent study assessed dentate gyrus neurogenesis in p75^NTR-/- ^mice and found a 50% decrease in the number of BrdU-positive cells using a 36 h BrdU labeling protocol followed by cell counts at the two week time point [24]. At the six-week time point, there was no difference in wildtype versus mutant mice in the number of cells co-labeled by BrdU and the neuronal marker NeuN. These observations raised the possibility that p75^NTR ^might be required for dentate progenitor proliferation. However, the key questions of whether p75^NTR ^is expressed by proliferating progenitors, whether p75^NTR ^is expressed by non-neuronal cells and whether genesis of non-neuronal cells is altered in p75^NTR ^mutant mice remain to be addressed.

In the present study we tested the hypothesis that p75^NTR ^is expressed by adult dentate gyrus progenitor cells expressing neuronal and/or glial markers and is required for their proliferation and differentiation into neurons, glia and/or other cells types.

## Results

### p75^NTR ^is present in SGZ progenitor cells

**Figure 1:**
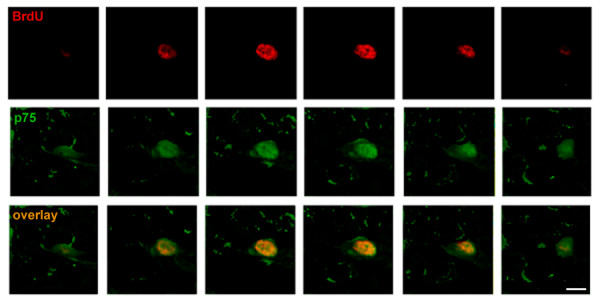
**p75^NTR ^expression in BrdU-positive SGZ cells, 10 hours post-BrdU injection**. Dentate gyrus coronal sections harvested from p75^NTR ^(+/+) mice 10 hours after a single BrdU injection were co-immunostained with antibodies against BrdU (red) and p75^NTR ^(green). Confocal microscope Z-stacks were collected at 40 × magnification with an interval of 1 μm between planes with a total of 6 image planes collected from each section. The typical appearance of SGZ fields is shown here. Scale bar (lower right panel): 2 μm.

The presence of p75^NTR ^in proliferating cells within the SGZ was assessed by quantitating cells co-expressing BrdU and p75^NTR^. Animals underwent a single injection of BrdU (300 mg/kg body weight) followed by sacrifice 10 h later [25]. SGZ progenitors demonstrate a cell cycle of ~14-16 hours [25] and become post-mitotic 3 days after their initial division [26]. As shown in Figure 1, confocal imaging identified SGZ cells expressing both p75^NTR ^and BrdU consistent with p75^NTR ^expression in proliferating progenitor cells. Within these cells, p75^NTR ^signal was evident in both a nuclear and cytoplasmic distribution (also see Additional file 1). Previous studies applying p75^NTR ^antibodies for assessment of SVZ progenitors and striatal neurons found either membrane-associated staining or diffuse intracellular staining [21,23,27].

**Figure 2:**
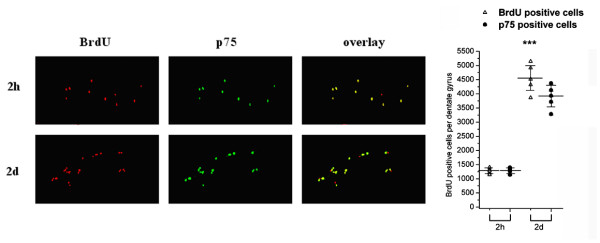
**p75^NTR ^expression in BrdU-positive SGZ cells, 2 hours and 2 days post-BrdU injection**. Dentate gyrus coronal sections harvested from p75^NTR ^(+/+) mice 2 hours or 2 days after a single BrdU injection were co-immunostained with antibodies against BrdU (red) and p75^NTR ^(green). Stereological estimates (as described in Methods) were used to quantitate the total number of BrdU-positive cells per dentate gyrus (open triangles) and BrdU-positive cells expressing p75^NTR ^(closed circles) (*** p < 0.001, Student-Newman-Keuls test after ANOVA, n = 6 mice with right and left dentate gyri averaged for each mouse). Scale bar: 200 μm.

In a second series of studies, animals received a single administration of BrdU at the same dose and were harvested at 2 h and 2d time points. At the first time point, numbers of BrdU-positive cells were relatively low and increased by approximately 3.5-fold by 2d (Figure 2). At the first time point essentially all of the BrdU-positive cells expressed p75^NTR ^while at 2d, some 80% expressed p75^NTR^.

### Dentate gyrus newborn cells express p75^NTR ^and neuronal or astrocytic markers

**Figure 3:**
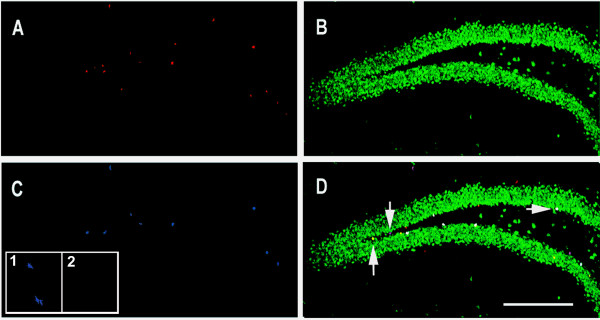
**Confocal image analysis of BrdU, NeuN and p75 ^NTR ^co-immunostaining**. Coronal sections of the dentate gyrus derived from a p75 ^NTR ^(+/-) mouse were examined. Tissue was obtained one day following the 6 day BrdU injection protocol. **(A) **BrdU antibody (red) detects cells primarily located in the SGZ and hilus. **(B) **NeuN staining (green) demonstrates the characteristic pattern of granule cell layer neurons in the dentate gyrus along with individual cells apparent in the subgranular zone and hilus. **(C) **p75 ^NTR ^staining (blue) identifies cells in the SGZ and hilus. **(D) **Overlay of images shown in A-C reveals cells co-labeled with BrdU, NeuN and p75 ^NTR ^(white, horizontal arrow); BrdU and p75 ^NTR ^(pink, up-down arrow); BrdU alone (red); BrdU and NeuN (yellow, down-up arrow). Scale bar: 300 μm. Inset in figure C: high magnification of p75^NTR ^staining in p75^NTR ^(+/+) **(1) **and (-/-) **(2) **mice.

**Figure 4:**
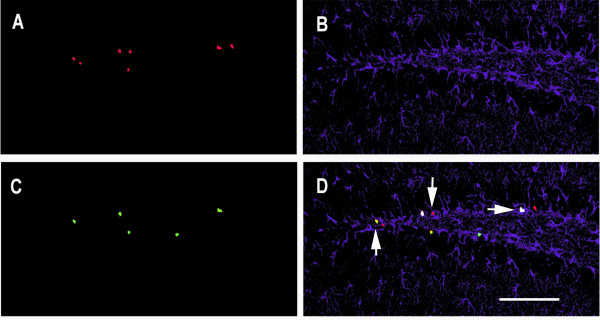
**Confocal image analysis of BrdU, GFAP and p75^NTR ^co-immunostaining**. Coronal sections of the dentate gyrus derived from a p75 ^NTR ^(+/-) mouse ware examined. Tissue was obtained one day following the 6 day BrdU injection protocol. **(A) **BrdU antibody (red) detects cells primarily located in the SGZ and hilus. **(B) **GFAP staining (purple) demonstrates a characteristic fibrillar pattern of astrocytes in the dentate gyrus. **(C) **p75 ^NTR ^staining (green) identifies cells in the SGZ. **(D) **Overlay of images shown in A-C reveals cells co-labeled with BrdU, GFAP and p75 ^NTR ^(white, horizontal arrow); BrdU and p75 ^NTR ^(yellow, down-up arrow); BrdU and GFAP (pink, up-down arrow); GFAP and p75 ^NTR ^(cyan) and BrdU alone (red). Scale bar: 100 μm.

P75^NTR ^(+/-) mice were used to evaluate whether newborn cells in the adult dentate gyrus expressing p75^NTR ^also express neuronal or astrocytic markers. Mice were injected daily for 6 days with BrdU (50 mg/Kg body weight), and the proportions of cells co-expressing BrdU and p75^NTR ^that also expressed NeuN or GFAP were measured (Figures 3 and 4). Following the 6d BrdU course, most of the dentate gyrus BrdU-positive cells were found to express p75^NTR^. A large proportion of BrdU/p75^NTR^-co-expressing cells also labeled with the NeuN neuronal marker (Figure 3D). A smaller proportion of BrdU/p75^NTR^-co-expressing cells were labeled with the GFAP astrocytic marker (Figure 4). As shown in the inset in Figure 3C, immunostaining with p75^NTR ^antibody of sections derived from p75^NTR ^-/- mice showed no significant signal and thus verified the specificity of the antibody. These findings suggest that a relatively large proportion of newborn p75^NTR^-positive cells differentiate into neurons with a smaller proportion becoming astrocytes.

### Proliferation of dentate gyrus progenitor cells in p75^NTR ^(+/+) and (-/-) mice

**Figure 5:**
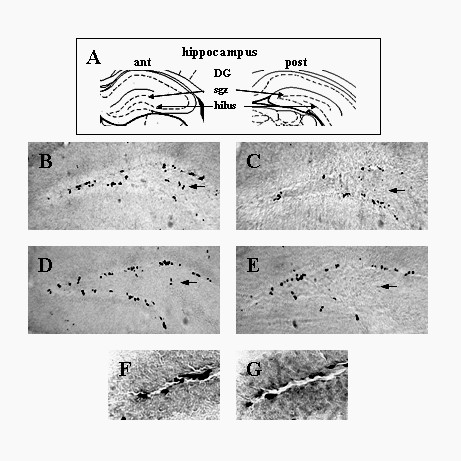
**Light microscopy analysis of BrdU-positive cells in p75^NTR ^(+/+) and (-/-) dentate gyrus**. Coronal sections of the dentate gyrus harvested from p75 ^NTR ^(+/+) **(B, D) **and p75 ^NTR ^(-/-) **(C, E) **transgenic mice were examined. **(A) **Coronal schematic of anterior and posterior dentate gyrus (modified from [54]). In p75 ^NTR ^(+/+) tissues (B and D), BrdU-positive cells are found in a characteristic arrangement in the SGZ adjacent to the dentate gyrus in both anterior (B) and posterior (D) sections. Labeled cells are also present in the hilar regions (arrows). In p75 ^NTR ^(-/-) mice (C and E) an apparent decrease in the number of BrdU-positive cells was found in both anterior (C) and posterior (E) regions. Labeled cells are absent in the hilar regions in p75^NTR ^(-/-) (arrows in C and E). (**F, G) **Higher magnification of labeled SGZ cells demonstrates characteristic aggregation of progenitor cells in p75^NTR ^(+/+) sections with less aggregate apparent in (-/-) sections. Scale bar 25 μm. Abbreviations: ant: anterior; post: posterior; sgz: subgranular zone; DG: dentate gyrus. Scale bar: 100 μm.

**Figure 6:**
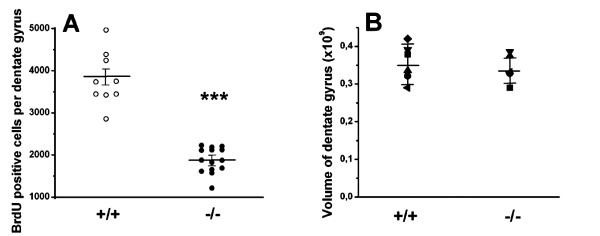
**Quantification of BrdU-positive cells in p75^NTR ^(+/+) and (-/-) mice**. Dentate gyrus volume and numbers of BrdU-positive cells were measured in coronal sections harvested from p75 ^NTR ^(+/+) and (-/-) mice. Tissues were obtained one day following the 6-day BrdU injection protocol. **(A) **Cell counts (as determined by stereological estimates (as described in Methods) in p75 ^NTR ^(-/-) mice (n = 14 mice) were significantly decreased compared to those in p75 ^NTR ^(+/+) mice (n = 9). *** *p *< 0.001 between groups, using Student-Newman-Keuls test after ANOVA. For each mouse the cell counts in right and left dentate gyri were averaged. **(B) **No difference in volume of the granular cell layer was detected between p75 ^NTR ^(+/+) and (-/-) mice (mean ± SE; n = 6 mice per group).

To determine whether p75^NTR ^plays in role in dentate gyrus progenitor proliferation and/or differentiation, p75^NTR ^(+/+) and (-/-) mice underwent the 6d BrdU injection protocol and the above proliferation and differentiation markers were assessed at the 1 day time point. Morphological assessment revealed a decreased number of BrdU-positive cells in p75^NTR ^(-/-) mice (Figure 5A-E). In (-/-) mice, BrdU-positive cells were more diffusely distributed compared to the aggregates of BrdU-positive cells that are typically seen in p75^NTR ^(+/+) and other wildtype mice [28,29] (Figure 5F,G). Quantitative stereological analyses (as described in Methods) demonstrated that in p75^NTR ^(-/-) mice the number of BrdU-positive cells was decreased by 59% (p < 0.001) relative to control mice (Figure 6A). The lack of any overlap between the values obtained from the (+/+) (n = 9 mice) and (-/-) (n = 14 mice) was consistent with a fully penetrant effect of the mutant genotype and thus further pointed to the significance of the effect of the (-/-) genotype on progenitor proliferation. This decrease in the number of BrdU-positive cells in p75^NTR ^(-/-) mice was not associated with a decrease in dentate gyrus volume (Figure 6B), thus consistent with a deceased in progenitor number and density per volume.

### Dentate gyrus progenitor differentiation in p75^NTR ^(+/+) and (-/-) mice

**Figure 7:**
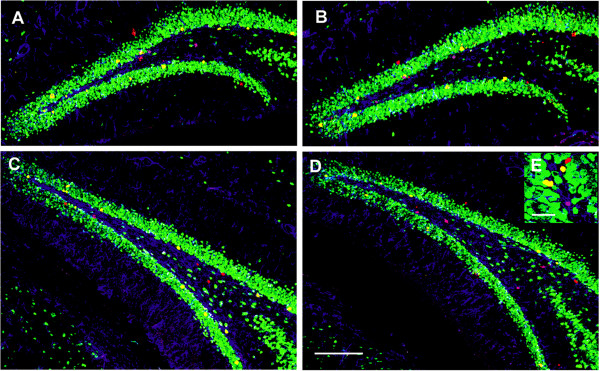
**Confocal image analysis of BrdU, NeuN and GFAP co-immunostaining in p75^NTR ^(+/+) and (-/-) mice**. Coronal sections of the dentate gyrus were co-immunostained with antibodies directed against BrdU (red), NeuN (green) and GFAP (blue). Sections harvested from p75 ^NTR ^(+/+) (**A, **anterior; **C, **posterior) and p75 ^NTR ^(-/-) (**B**, anterior; **D**, posterior) mice one day following the 6-day BrdU protocol were examined. Sections from p75 ^NTR ^(-/-) mice (B-D) demonstrate an apparent decrease in the number of SGZ BrdU-positive neurons colocalizing with NeuN marker (yellow) compared to wildtype mice (A-C). **(E)**: Insert: cells staining for BrdU and NeuN (yellow); BrdU and GFAP (pink); BrdU (red); NeuN (green) and GFAP (blue). Scale bar, A-D: 300 μm and E: 15 μm.

**Figure 8:**
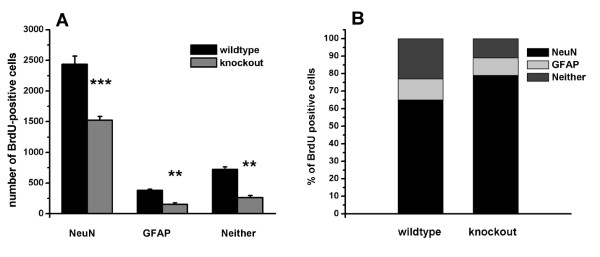
**Quantitative analysis of NeuN and GFAP markers expressed by BrdU-positive cells**. Coronal sections of the dentate gyrus were co-immunostained with antibodies directed against BrdU, NeuN and GFAP as demonstrated in Figure 7. The numbers of BrdU-positive cells co-labeling with NeuN, GFAP or neither marker were determined by stereological estimates (as described in Methods). For each mouse the cell counts in right and left dentate gyri were averaged. **(A) **A significant decrease in the number of BrdU-positive cells co-expressing NeuN, GFAP and neither marker was found in p75 ^NTR ^(-/-) sections. **(B) **The percentage of BrdU-positive cells expressing NeuN, GFAP and neither marker are indicated. BrdU-positive cells co-labeling with NeuN demonstrated a significant increase from 66% in p75^NTR ^(+/+) mice to 81% in (-/-) mice. *** *p *< 0.001, ** *p *< 0.01, between groups, using Student-Newman-Keuls test after ANOVA (n = 6 (+/+) and 8 (-/-) mice).

To determine whether the decrease in progenitor proliferation in p75^NTR ^(-/-) dentate gyrus was associated with altered differentiation of progenitors into neurons or glia, sections from the above mice were assessed for co-labeling of BrdU-positive cells with NeuN and GFAP. Confocal microscopic images of cells triple-labeled for BrdU, NeuN and GFAP confirmed the expected presence of newborn cells expressing NeuN or GFAP (Figure 7A-D). In p75^NTR ^(-/-) mice, stereological estimates (as described in Methods) (Figure 8A) of BrdU-positive cells revealed a 35% decrease in the number cells co-expressing NeuN, a 60% decrease in the number cells co-expressing GFAP and a 64% decrease in the number of cells expressing neither marker, with the decrease reaching statistical significance for all three categories. Assessment of the proportions of BrdU-positive cells within these three marker categories (Figure 8B) demonstrated that the proportion of BrdU-positive cells co-labeling with NeuN increased from 66% in p75^NTR ^(+/+) mice to 81% in (-/-) mice. In contrast, the proportions of BrdU-positive cells co-labeling with GFAP or neither marker decreased in p75^NTR ^(-/-) mice. Thus, in p75^NTR ^(-/-) mice, dentate gyrus cellular proliferation as indicated by BrdU labeling is considerably decreased (by 59%) and this decrease includes significantly reduced numbers of cells with neuronal, glial and neither markers. Within this context there is a concomitant modest increase in the proportion of BrdU-positive cells expressing NeuN.

## Discussion

This study demonstrates three key findings relevant to the identification of receptors regulating neurogenesis: first, newborn cells in the adult dentate gyrus, labeling with either neuronal or glial markers, express p75^NTR ^during early stages of proliferation; second, the number of SGZ newborn cells is significantly reduced in p75^NTR ^(-/-) mice; and third, the numbers of cells in all three categories, those expressing neuronal, glial or neither marker, are significantly reduced in p75^NTR ^(-/-) mice.

The 59% decrease in the number of newborn SGZ cells in p75^NTR ^(-/-) mice found here is similar to the findings of Catts et al.[24], using a 36 hour BrdU oral labeling regime and a 2-week chase period [24], thereby further supporting a role of p75^NTR ^in dentate progenitor proliferation. The prior study also found a 50-60% reduction in the number of BrdU- NeuN-positive cells at the 2-week time point while the present study detected a 35% decrease in the number of BrdU- NeuN-positive cells at the 1 day time point following the 6-day BrdU labeling protocol. Hence, our study further supports a role for p75^NTR ^in neurogenesis. This effect could be caused by p75^NTR ^promoting survival and/or differentiation of cells undergoing neuronal differentiation. In contrast to the Catts et al study, we found no decrease in dentate gyrus volume. A lack of effect on dentate volume has also been noted in other studies in which SGZ progenitor proliferation and/or neurogenesis is altered [29-31]. In addition to the difference in time points examined, the prior study applied an oral BrdU dose of 650 mg/kg/day compared to the present IP dose of 50 mg/kg/day. While a number of factors are likely to contribute to the difference in findings in terms of dentate volume loss between the studies, it is of interest to note that high BrdU concentrations have been associated with neuronal death [32].

In addition to the effect of p75^NTR ^on neuron formation, a related critical question is its role in the genesis of non-neuronal cells. In the present study, the GFAP glial maker was applied and revealed that in p75^NTR^(-/-) mice, there were 60% and 64% decreases in the total number of newborn cell labeled with GFAP or neither marker, respectively. Thus, in p75^NTR^(-/-) mice the proportion of newborn cells expressing the NeuN marker is increased while the proportion without this marker is decreased. These studies introduce the important concept that while p75^NTR ^appears to contribute to neurogenesis, it might make an even greater contribution to formation of various populations of non-neuronal cells. Thus p75^NTR ^cannot be linked specifically to neurogenesis, but instead appears to play a broader role in dentate cell genesis.

The identification of a role for p75^NTR ^in dentate gyrus progenitor cell production is consistent with the emerging picture of p75^NTR ^regulating cell cycle mechanisms as well as regulating the proliferation and/or differentiation of progenitors or embryonic stem cells in cell culture models and in the SVZ *in vivo *[16-18,22]. These findings are also consistent with other studies in p75^NTR ^(-/-) mice in which a greater number of sympathetic neurons are present in early development while at later stages a decrease in the number of mature sympathetic neurons was found, suggesting that during development, p75^NTR ^might first induce proliferation and later apoptosis [12,33,34]. Similarly, during embryonic development, p75^NTR ^appears to participate in the early stages of hippocampal cell proliferation, but at later stages induces death of neurons during maturation [12,35]; and finally at the adult stage is expressed at low levels [11,33,36].

While alterations of intrinsic signaling mechanisms in p75^NTR ^(-/-) progenitors are likely to account for the decrease in neurogenesis observed here, it is also possible that factors extrinsic to progenitor cells contribute. For example, lesions in the entorhinal cortex, hippocampal CA_1 _and CA_3 _subregions or dentate gyrus induce an increase in dentate neurogenesis [37-40]. Decreased cholinergic input into the dentate gyrus has also been associated with decreased neurogenesis [41]. Interestingly, the p75^NTR ^(-/-) mice used in the present study and other strains carrying the same mutation were shown to have increased dentate cholinergic innervation [42,43]. Thus, the p75^NTR ^(-/-) mice employed here would be expected to have increased, rather than decreased, neurogenesis if the extrinsic factor of cholinergic innervation played a predominant role.

Studies demonstrating age-related impairments in hippocampal neurogenesis along with recent work showing that brain proNGF levels increase with age [44] raise the possibility that proNGF might contribute to loss of newborn cells through its interaction with p75^NTR^. Recently developed p75^NTR ^small molecule ligands are able to promote pro-survival signaling and are also able to prevent proNGF-induced death [45]. Findings in the present study will encourage studies to establish whether p75^NTR ^small molecule ligands can modulate dentate gyrus cell production.

## Conclusions

Our results indicate that p75^NTR ^receptor plays a fundamental role in the generation of dentate newborn cells in the adult brain. Of particular interest are the novel findings that p75^NTR ^is expressed by BrdU-positive cells and that it is also expressed by newborn cells during the time periods in which they express neuronal or glial markers. An additional novel finding is that numbers of newborn neurons, as well as non-neurons, are decreased in p75^NTR^(-/-) mice pointing to a role for this receptor in the formation of neurons as well as non-neurons. These results provide a basis for examining the effects of recently developed p75^NTR ^small molecule ligands in dentate cell formation.

## Methods

### Animals

Animal studies were performed according to National Institutes of Health guidelines under an approved local protocol from the San Francisco VA Medical Center. Two sets of animals were used. Balb/c wildtype mice, and mice carrying a mutation in exon 3 of the p75^NTR ^gene [46], both purchased from the Jackson laboratory (Bar Harbor, ME). These mutant mice were created using 129 strain ES cells with subsequent breeding in a mixed 129/Balb/c background. Mutant mice were maintained in our colony via successive Balb/c backcrosses. Mice used in the present study were derived via a minimum of 6-8 Balb/c backcrosses resulting in estimated congenic Balb/c homogeneity of 97-99% [47]. Genotyping was conducted using RT-PCR as previously described [42]. p75^NTR ^(+/-) littermate crosses were used to generate p75^NTR ^(+/+) and (-/-) littermates. All studies were limited to littermate comparisons and mice were studied at 3 months of age unless otherwise indicated. In each genotype group, approximately equivalent numbers of male and female mice were included. Animals were housed in standard conditions with four or five mice per cage.

### BrdU injections and tissue preparation

Bromodeoxyuridine (BrdU; Sigma, St. Louis, MO) was dissolved in sterile 0.9% NaCl and filtered. Mice received i.p. injections using the dosages and schedules described in Results. Animals underwent transcardial perfusion with 4% paraformaldehyde in phosphate buffer. Brains were harvested, stored in fixative overnight, transferred into 30% sucrose and stored overnight at 4°C. Coronal sections (50 μm thick) were cut on a sliding freezer microtome and stored at 4°C in 0.1 M phosphate buffer containing 0.005% azide. In order to denature DNA for BrdU immunohistochemistry, free-floating sections were incubated in 50% formamide/50% 2 × SSC (0.3 M NaCl/0.03 M sodium citrate) at 37°C for 2 hours, washed in 2 × SSC, incubated in 2N HCl for 30 minutes at 37°C and rinsed in 0.1 M borate buffer (pH 8.5) for 15 minutes.

### Immunohistochemistry

Sections were treated with 0.6% H_2_O_2 _to block endogenous peroxides, rinsed in phosphate buffer 2 × 10 min and then incubated in blocking solution (0.1 M phosphate buffer, 0.1% triton X-100, 2% normal serum and BSA 1 g/l) for 1 h. Sections were then incubated with mouse monoclonal BrdU antibody (Boehringer Mannheim, Indianapolis, Indiana; 1:400) overnight at 4°C followed by incubation with biotinylated sheep anti-mouse IgG secondary antibody (1:200; Amersham, Piscataway, New Jersey) for 1 h. ABC reagent (50 μl/5 ml; Vectastain Elite, Vector Laboratories, Burlingame, California;) was applied for 2 h. Diaminobenzidine (Fast-DAB; Sigma) was used as chromogen. Stained cells were visualized under light microscopy using differential interference contrast (Zeiss Axoplan 2). Images were acquired using a Pixera digital camera and processed with Adobe Photoshop 5.5. Only general contrast enhancements and color level adjustments were carried out; images were not otherwise digitally modified.

### Immunofluorescence

After pretreatment for BrdU, sections were kept in blocking solution for 2 h. They were then incubated for 24 h at 4°C with primary antibodies diluted in 0.1 M TBS containing 0.1% triton and 5% donkey serum (TBS-T). Antibodies were directed against the following antigens: BrdU (rat monoclonal, 1:500; Accurate Scientific, Westbury, New York); p75^NTR ^extracellular domain (rabbit monoclonal, cat# 05-446, lot# 22972, 1:400; Chemicon, Temeluca, California, now a part of Millipore, Massachusetts), NeuN (mouse monoclonal, 1:800; Chemicon) and GFAP (rabbit polyclonal, Dako, 1:1000; Glostrup, Denmark, and mouse monoclonal, Boehringer Mannheim, 1:1000, Indianapolis, USA). Secondary antibodies raised in donkey (Cy3 for detecting BrdU; FICT for p75^NTR ^or NeuN, and Cy5 for GFAP or p75^NTR^; all from Jackson ImmunoResearch; West Grove, Pennsylvania; 6 μg/ml) were applied for 2 h at room temperature. Sections were washed, mounted, and coversliped in mounting medium for fluorescence (Vectashield, Vector Laboratories, Burlingame, California). Fluorescent signal was detected using a confocal microscope (Leica Laser Confocal TCS SP) and the images were processed with Adobe Photoshop. Only general contrast enhancements and color level adjustments were carried out; images were not otherwise digitally modified.

### Quantification of BrdU-labeled cells

Under light microscopy (using mercury lamp settings on the Leica Laser Confocal TCS SP microscope), numbers of DAB-stained cells within the subgranular layer located within one cell diameter of the granule cell layer boundary were determined using the optical disector principle in which the uppermost focal plane of each section was not counted [48,49]. The number of DAB-stained cells in the subgranular zone was counted in every fourth section within a series of 50 μm coronal sections extending throughout the rostra-caudal axis of the granular cell layer. Cell counts were restricted to the top 15 μm of each section where immunostaining was optimal (see below). The first section to be counted was randomly selected out of the first four cut sections and 7-9 sections were assessed in order to span each dentate gyrus. The disector height was set at 6 μm [50] and from each section, 7-8 z-planes were assessed to count BrdU-positive cells. The estimated final mounted section mean thickness was 36.46 μm (consistent with post mounting dehydration); with a coefficient of variation of 0.041. Guard zones (distance between the section surface and the optical disector) were 4 μm on top and bottom sides. For each section, the entire dentate gyrus area was used to evaluate the BrdU-positive cells, thus no sampling was applied. For performing cell counts with either DAB or fluorescent staining, a 40×, Plan-Apochromat, NA: 1.25, oil immersion objective was applied. BrdU-positive cells were also present in the hilar area (arrows in Figure 5B-E), but these cells were not counted since they likely represent endothelial and other non-neuronal cells [28,29].

### Quantification of granular cell reference volume

Granular cell reference volumes were estimated using previously established protocols and Cavalieri's direct estimator [48,49,51]. Serial coronal sections were imaged using the Leica TCS-SP confocal microscope with a multiband confocal imaging spectrophotometer. For each section, the GCL area was outlined using 20 × objective images (20×, Plan-Apochromat, NA: 0.7, multiple immersion) and analyzed with Scion Beta 4.0.2 (NIH) software. GCL total volume was calculated from V = ∑A × T × 4 where ∑A is the sum of area measurements, T is the section thickness (50 μm) and 4 is the periodicity of the section sample. For volume determination, the coefficient of error was calculated to be 0.04 (the standard deviation of section area values divided by the square root of the estimated mean, Glaser and Wilson [52]). For volume studies, 6 animals from each genotype group were randomly selected, right and left dentate gyri were measured and for each mouse the two values were averaged.

### Colocalization of BrdU and cell phenotype markers

Colocalization analysis was performed using the Leica Laser Confocal TCS SP microscope. Sections were optically sliced in the Z-axis at 4 μm intervals and fluorescent images were acquired, one per each marker. In early studies, we determined that immunostaining signal was robust without an observable decline through Z-axis distances of approximately 20 μm from the section surface and thereafter began to decrease. Therefore, analysis was restricted to the top 15 μm of each section where immunostaining was optimal. In each section the number of BrdU-positive cells associated with markers for NeuN or GFAP was counted as described by Peterson [53]. For each dentate gyrus, 7-9 sections were assessed (the number required to fully span each dentate gyrus) and each BrdU-positive cell were assessed for phenotype markers. For each mouse, data from the right and left gyri were averaged. The proportions of BrdU-positive cells associated with NeuN and GFAP markers were calculated. Images obtained from individual optical slices were imported to Adobe Photoshop 5.5 for composition of figures.

### Statistics analysis

The data are expressed as mean values ± standard error of mean (SEM) from *n *independent experiments. Statistical analyses were performed using one-way analysis of variance (ANOVA) with number of positive cells as a factor, followed by Student-Newman-Keuls post hoc comparisons or Student's t test, when required.

## Authors' contributions

RB and FL each contributed to the conceptualization, design, execution and analysis of these studies. Both contributed to the manuscript preparation.

## Supplementary Material

Additional file 1 **Confocal 3D reconstructed imaging of p75^NTR ^expression in BrdU-positive granular cells**. Dentate gyrus coronal sections harvested from three p75^NTR ^+/+ mice were co-immunostained with antibodies against p75^NTR ^(green) and BrdU (red). A representative confocal 3D-reconstructed, merged image of a SGZ cell demonstrates nuclear BrdU signal with p75^NTR ^signal evident in cytoplasmic and nuclear areas. Orthogonal images demonstrate x - z (top) and y - z (right) planes. Scale bar: 5 μm. Click here for file
