# Supplementary figures and images for: The p75 neurotrophin receptor is expressed by adult mouse dentate progenitor cells and regulates neuronal and non-neuronal cell genesis
Source: BMC Neurosci. 2010 Oct 20;11:136. doi: 10.1186/1471-2202-11-136 (PMC2987811; doi:10.1186/1471-2202-11-136)

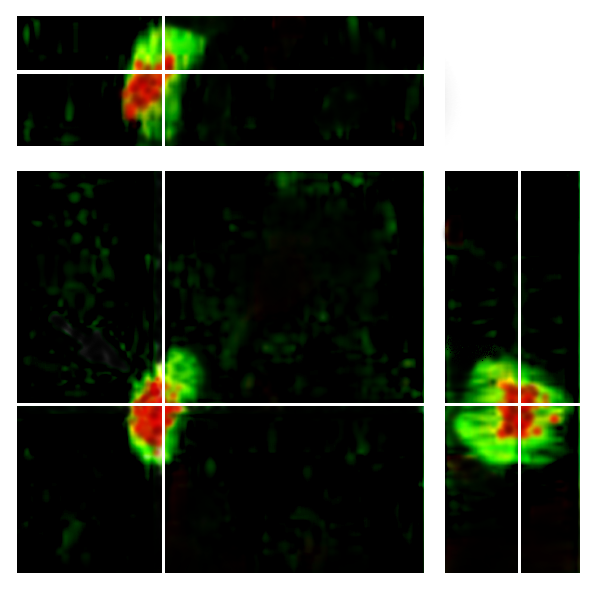

Supplement: Additional file 1 — Confocal 3D reconstructed imaging of p75NTR expression in BrdU-positive granular cells. Dentate gyrus coronal sections harvested from three p75NTR +/+ mice were co-immunostained with antibodies against p75NTR (green) and BrdU (red). A representative confocal 3D-reconstructed, merged image of a SGZ cell demonstrates nuclear BrdU signal with p75NTR signal evident in cytoplasmic and nuclear areas. Orthogonal images demonstrate x - z (top) and y - z (right) planes. Scale bar: 5 μm. [file 1471-2202-11-136-S1.TIFF]
